# Beta-tricalcium phosphate granules improve osteogenesis *in vitro* and establish innovative osteo-regenerators for bone tissue engineering *in vivo*

**DOI:** 10.1038/srep23367

**Published:** 2016-03-22

**Authors:** Peng Gao, Haoqiang Zhang, Yun Liu, Bo Fan, Xiaokang Li, Xin Xiao, Pingheng Lan, Minghui Li, Lei Geng, Dong Liu, Yulin Yuan, Qin Lian, Jianxi Lu, Zheng Guo, Zhen Wang

**Affiliations:** 1 Department of Orthopaedic, Xijing Hospital, Fourth Military Medical University, Xi’an, Shaanxi, 710032, P.R. China; 2 Department of Oncology, Xijing Hospital, Fourth Military Medical University, Xi’an, Shaanxi, 710032, P.R. China; 3 State Key Laboratory for Manufacturing Systems Engineering, Xi’an Jiaotong University, Xi’an 710054, China; 4 Shanghai Bio-Lu Biomaterials Co., Ltd., Shanghai, 201114, P.R. China

## Abstract

The drawbacks of traditional bone-defect treatments have prompted the exploration of bone tissue engineering. This study aimed to explore suitable β-tricalcium phosphate (β-TCP) granules for bone regeneration and identify an efficient method to establish β-TCP-based osteo-regenerators. β-TCP granules with diameters of 1 mm and 1–2.5 mm were evaluated *in vitro*. The β-TCP granules with superior osteogenic properties were used to establish *in vivo* bioreactors, referred to as osteo-regenerators, which were fabricated using two different methods. Improved proliferation of bone mesenchymal stem cells (BMSCs), glucose consumption and ALP activity were observed for 1–2.5 mm β-TCP compared with 1-mm granules (P < 0.05). In addition, BMSCs incubated with 1–2.5 mm β-TCP expressed significantly higher levels of the genes for runt-related transcription factor-2, alkaline phosphatase, osteocalcin, osteopontin, and collagen type-1 and the osteogenesis-related proteins alkaline phosphatase, collagen type-1 and runt-related transcription factor-2 compared with BMSCs incubated with 1 mm β-TCP (P < 0.05). Fluorochrome labelling, micro-computed tomography and histological staining analyses indicated that the osteo-regenerator with two holes perforating the femur promoted significantly greater bone regeneration compared with the osteo-regenerator with a periosteum incision (P < 0.05). This study provides an alternative to biofunctionalized bioreactors that exhibits improved osteogenesis.

A critical size bone defect is usually caused by high-energy trauma^1^, bone tumour, infection or congenital deformity. Critical size bone defects are often too large to be repaired by self-healing and will thus eventually result in limb length discrepancy, deformation and dysfunction^2^,^3^. Tissue engineering is considered a possible treatment for bone defects^4^. In traditional bone tissue engineering, autologous bone marrow cells are obtained from the patient, seed on a bioactive scaffold and cultivate in a bioreactor^5^. Considerable new bone growth has been achieved using these approaches in various *in vitro* bioreactors^6^,^7^,^8^. However, it is a major challenge to fabricate a bone substitute with the appropriate size and abundant activity^9^. *Ex vivo* bioreactors can closely mimic the *in vivo* organism, but few can recreate the microenvironment of a complex organism *ex vivo*. Consequently, interest is increasing in technologies in which the patient functions as the bioreactor, thus eliminating *ex vivo* bioreactor cultivation^10^. Bioactive scaffolds have been used to produce tissue-engineered bone in various parts of the bodies of animals or humans, including subperiosteal, muscle, fat and subcutaneous tissues^11^,^12^,^13^,^14^,^15^. Unfortunately, there is no optimal location that can provide all cells and signals required for bone regeneration, and many of these methods require the application of exogenous cells and growth factors^16^. Thus, identifying an appropriate site to construct an *in vivo* bioreactor that does not require exogenous cells and growth factors is of great importance.

Space-filling scaffolds provide a substrate on which new tissue can form and remodel. However, the scaffold is not only a passive support but also provides cells and signals that induce the development of new bone^17^. A wide range of scaffolds that are effective in tissue-engineered bone regeneration have been designed^18^,^19^,^20^,^21^,^22^,^23^. However, the scaffolds used in research and clinical applications are suboptimal, and improved scaffolds for *in vivo* bioreactors are needed. Several bioceramic materials, including β-tricalcium phosphate (β-TCP), hydroxyapatite (HA), and calcium sulfate, have been extensively used in orthopaedics as bone substitutes^24^. β-TCP is the most widely used due to the limitations of HA and calcium sulfate as bioceramic materials. The limited ability of HA to degrade and absorb within the body hinders the formation and remodelling of new bone^25^, potentially leading to permanent stress concentration and poor local stability^26^. By contrast, calcium sulfate is degraded and absorbed quite rapidly *in vivo*. This degradation often occurs prior to the formation of new bone, resulting in a discrepancy between new bone formation and material absorption. β-TCP scaffold materials are attractive as bone substitutes due to their biocompatibility, biological safety, virtually unlimited availability, ease of sterilization, and long shelf life^27^. β-TCP represents a good balance among absorption, degradation and new bone formation and can also preserve structural stability by releasing a large quantity of calcium (Ca^2+^) and sulfate (SO4^2−^) ions, indispensable inorganic salts for new bone formation^28^,^29^.

Different types of β-TCP have been developed in recent years. β-TCP granules appear adequate for clinical use, but pre-clinical studies of their potential osteogenic properties are lacking.

An optimal scaffold and suitable “regeneration factory”, that is, an *in vivo* site in the body, are of great importance for the advancement of tissue engineering research.

This research comprised two parts. First, β-TCP granules with sizes of 1 mm and 1–2.5 mm were evaluated to determine which size better promoted osteogenesis *in vitro*. Then, the β-TCP granules with superior osteogenic properties were used *in vivo* to establish *in vivo* bioreactors, termed osteo-regenerators. New Zealand rabbit femurs were chosen as the core of the osteo-regenerators. Two osteo-regenerators were established using two different methods. In one method, two penetrating holes were drilled in the femur; in the other, an incision was made in the periosteum. To retain the β-TCP granule scaffolds around the femur, two semicylindrical titanium alloy shields were generated using three-dimensional printing technology to fabricate the osteo-regenerator.

In this study, we hypothesized that β-TCP granules with diameters of 1–2.5 mm would better promote osteogenesis *in vitro*. The goal of the osteo-regenerators was to fabricate new bone in the absence of exogenous stem cells and growth factors and that the osteo-regenerator with holes that penetrated the femur would result in improved bone formation compared with the osteo-regenerator with the periosteal incision.

## Methods

### β-TCP granules

β-TCP granules with diameters of 1 mm and 1–2.5 mm were a gift from Shanghai Bio-lu Biomaterials Co., Ltd. We described the method of fabrication of the β-TCP granules previously^17^,^30^. The granules were irregular in shape, and each had interconnection diameters ranging from 70 to 200 μm. The porous structure of β-TCP was characterized using a scanning electron microscope (SEM, S-4800, Hitachi, Japan). The porosity and interconnections of the porous β-TCP were calculated using an analysis software package (Micro-view ABA 2.1.2; GE)^31^. Energy spectrum analysis system (HORIBA EMAX, EX-450, Japan) was performed to determine the composition and weight percentage of β-TCP granules.

### Semicylindrical porous titanium alloy shields

**Figure 1:**
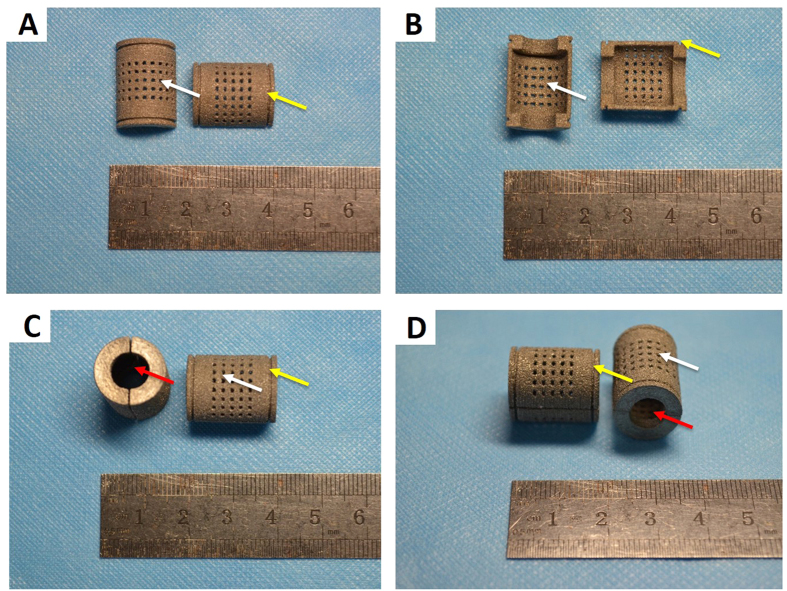
Images of the porous titanium alloy shields. (**A**) The white arrows indicate the small holes. (**B**) The yellow arrows indicate the grooves. (**C**) The red arrows indicate the large holes.

The semicylindrical porous titanium alloy (Ti6Al4V) structure was designed using commercial CAD software (Unigraphics NX, EDS). The CAD data for the structure were converted to STL data and imported into the three-dimensional printing machine. The samples were produced with high productivity and high accuracy. The titanium shields contained multiple 1-mm holes spaced at constant 1-mm intervals. There were two 1-mm-deep grooves instead of holes at either end of the outer surface of the titanium alloy. The two deep grooves ensured that the two porous titanium alloy semicylinders formed a cylinder secured by steel wires. The cylinders were hollow and had two large, 9-mm holes at the top and bottom. The diameter of these holes is equivalent to the diameter of the femur. The length and diameter of the titanium cylinder were 20 mm and 15 mm, respectively (Fig. 1).

### *In vitro* experiments

#### BMSC isolation and culture

Bone mesenchymal stem cells (BMSCs) were isolated from the tibiae and femurs of young, 4- to 6-week-old mice. The soft tissues were cleaned, and the epiphysis cartilage from both ends of the femurs and tibiae were removed. We used α-minimal essential medium (α-MEM) supplemented with 15% foetal calf serum, 100 pg ml^−1^ penicillin G, 500 pg ml^−1^ gentamicin and 0.3 pg d^−1^ Fungizone to flush out the BMSCs using a 23-gauge needle. The cell suspensions were cultured in the previously described medium in a humidified atmosphere of 95% air and 5% CO_2_ at 37 °C. The medium was changed after 24 hours to remove non-adherent cells. The remaining adherent cells were mainly BMSCs^32^. The medium was changed every other day thereafter. We used BMSCs from the third passage in our experiments after cultivation.

#### Cell proliferation of BMSCs with β-TCP granules

Cell proliferation was evaluated using cell counting kit-8 (CCK-8, Dojindo, Japan). BMSCs were incubated in twelve 24-well plates at a density of 4 × 10^4^ cells per well in the previously described medium. BMSCs were cultured with β-tricalcium phosphate (β-TCP) granules with sizes of 1.0 mm (group A) and 1.0–2.5 mm (group B) at the same weight of 30 mg per well. The proliferation rate of BMSCs was estimated using the CCK-8 assay on days 1, 4, and 7. After cultivation to these time points, the samples were transferred to new 24-well culture plates. CCK-8 solution with a 10% volume of the medium was then added to the wells, and the samples were incubated at 37 °C for 2 h. Then, 100 μL of the reaction solution was transferred to a new 96-well plate, and the optical density was measured at 450 nm by a microplate reader^33^.

In the *in vitro* experiments, group A refers to BMSCs cultured with 1.0-mm β-TCP granules, and group B refers to BMSCs cultured with 1.0–2.5 mm β-TCP granules.

#### Glucose consumption

The cellular utilization of D-glucose was analysed by spectrometry according to the protocol developed by Roche (D-glucose UV-method, Roche, Germany). Samples were collected during each medium change and diluted appropriately to remain within the assay detection range. The assay was performed following the manufacturer’s instructions, and the absorbance of each cuvette at 505 nm was measured using a spectrophotometer. Fresh complete medium and D-glucose control solution were assayed as references. Finally, the glucose concentration was quantified based on the difference in absorbance, and the glucose consumption was expressed as the average daily glucose reduction in the medium^34^.

#### ALP activity

To evaluate differentiation in the early phase, the alkaline phosphatase (ALP) activity of each sample was measured at 2, 4, 6 and 8 days of cultivation. The ALP activity was determined by a colorimetric assay using an ALP reagent containing p-nitro-phenyl phosphate (p-NPP) as the substrate. The optical density of the generated p-nitrophenol was measured at 405 nm using a spectrophotometer. All samples were assayed in triplicate. The protein concentration was estimated using a protein assay kit (Pierce, Rockford, IL). The ALP activity was expressed as μmol/h/mg of protein/g of β-TCP granule scaffold.

#### Quantitative real-time PCR

BMSCs were seeded on samples at a density of 4 × 10^4^/well in 24-well culture plates. After treatment with osteogenic medium for 7 and 14 days, the expression levels of ALP, collagen type-1 (Col-1), osteocalcin (OCN), runt-related transcription factor-2 (Runx2) and osteopontin (OPN) were determined to evaluate the differentiation of the cultured cells. Total RNA was extracted from the cells cultured on the samples at each time point using TRIzol reagent (Invitrogen Life Technologies) and subsequently converted to cDNA using Prime Script RT Master Mix (Takara). The RT-PCR reactions were performed using SYBR Premix Ex Taq II (Takara) on a CFX96 PCR System (Bio-Rad). The concentration of RNA was determined by measuring the optical absorbance of the extract at 260 nm. The housekeeping gene GAPDH was used as an internal control. The primer sequences are shown in Table 1.

#### Western blot analysis

On days 7 and 14, the BMSCs co-cultured with the β-TCP granules with a diameter of 1 mm (Group A) or 1–2.5 mm (Group B) in six-well plates were washed twice with PBS. The BMSCs cultured without β-TCP granules were used as the control group. Cellular proteins were extracted in lysis buffer containing 20 mmol Tris (pH 7.4), 150 mmol NaCl, 1 mmol EDTA, 1 mmol EGTA, 1% Triton, 0.1% SDS and 1% protease inhibitor cocktail. Equal amounts (30 μg of protein) of protein were separated on 10% or 15% SDS-polyacrylamide gels in a minigel apparatus (Mini-PROTEAN II, Bio-Rad) and transferred electrophoretically to nitrocellulose membranes. The membranes were blocked with 5% milk in Tris-buffered saline (TBS) before incubation with antibodies overnight at 4 °C. The membranes were incubated for 1 hour with a horseradish peroxidase (HRP)-conjugated secondary antibody (1:5,000). After immunoblotting, the films were scanned, and the intensity of the immunoblot bands was quantified on a Bio-Rad Calibrated Densitometer. β-actin was used as the loading control.

### *In vivo* experiment

#### Surgical procedure

**Figure 2:**
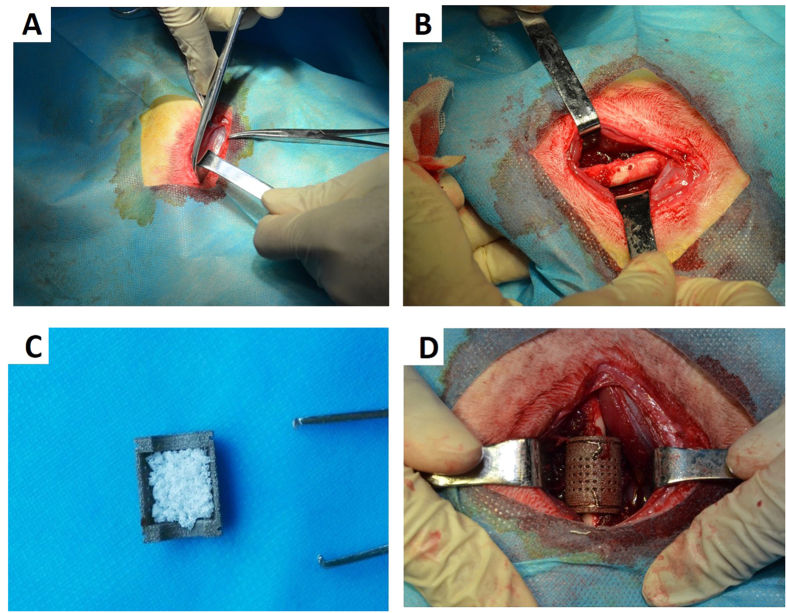
Images of the surgical procedure. (**A**) Shows the longitudinal incision in the rabbit femur. (**B**) Shows the two perforating holes, which were 1 mm in diameter, in the femoral shaft. (**C**) Displays a titanium shield filled with 1–2.5 mm β-TCP granules. (**D)** Shows the entire osteo-regenerator fixed around the femur of a rabbit.

Thirty-six New Zealand white rabbits with an average weight of 3 ± 0.5 kg were randomly divided into two groups according to the implant method. The rabbits were narcotized by intravenous injection of 0.5 mg kg^−1^ acepromazine (Calmivet-Vetoquinol) and 10 mg kg^−1^ ketamine. A longitudinal incision approximately 4.0 cm in length was made through the full thickness of the femur skin, and the muscles were bluntly separated by forceps to expose the femur. In group I, we drilled two holes with a diameter of 1 mm that perforated the femoral shaft; the holes were separated by 5 mm. Afterward, one titanium shield filled with 1–2.5 mm diameter β-TCP granules was placed below the femur, with the two holes situated precisely in the middle of the shield. Next, another titanium shield filled with β-TCP granules was inserted and properly aligned with the previous shield. Two steel wires were used to fasten the two semicylindrical titanium shields around the shaft of the femur (Fig. 2). In group II, we made an incision in the periosteum of the femur instead of drilling holes in the femur itself. After sufficient irrigation with normal saline, the wound was closed layer by layer. Antibiotics were intramuscularly injected postoperatively twice with a 3-day interval. The rabbits were sacrificed by intravenous injection with an overdose of anaesthetic at 4, 8 and 12 weeks post-operation, and the implants were harvested and soaked in 80% ethanol for further analysis.

In all *in vivo* experiments, in Group I, we drilled two 1-mm-diameter holes that perforated the femoral shaft to construct the osteo-regenerator. By contrast, in group II, we made an incision in the periosteum of the femur to construct the osteo-regenerator.

The surgery and treatment of rabbits were performed strictly according to the regulations and laws of our country and in accordance with the Standing Committee on Ethics in China. The animal experiments were approved by the Fourth Military Medical University Committee on Animal Care and were conducted at the Laboratory for Animal Research of the Research Institute of Orthopaedics at XiJing Hospital affiliated with the Fourth Military Medical University in China. All experiments involving animals were performed in accordance with the approved guidelines.

#### Fluorochrome labelling

Sequential fluorochrome markers were administered to monitor the mineralization process of new bone formation. At 2 weeks or 3 days prior to sacrifice, the animals were injected with tetracycline (50 mg/kg) or calcein (25 mg/kg), respectively. After the animals were sacrificed, samples were obtained and fixed in 80% ethanol for two weeks before fluorescence analysis.

#### Micro-computed tomography evaluation

Samples were extracted (n = 6 in each group), fixed in 80% ethanol for two weeks, and then scanned by micro-computed tomography (Micro-CT) (Inveon, Siemens, Germany). Approximately 1600 projections of 1024^2^ pixels were acquired for each tomogram. The X-ray source voltage was set at 80 kV with the beam current at 200 mA using filtered Bremsstrahlung radiation. Specimens were reconstructed and evaluated using 3D analysis software (Microview, GE Healthcare, Canada) in the ROI. The bone volumes (mm^3^) in the osteo-regenerators were analysed using the instrument-provided software based on the different computed tomography (CT) values of the β-TCP granule scaffold, titanium shield and bone^35^,^36^.

#### Histological examination and quantitative histological analysis

After micro-CT scanning, the samples were dehydrated in a graded series of ethanol (80–100%) and cleared with toluene. All specimens were then embedded in methylmethacrylate. After polymerization, approximately 70-mm-thick serial transverse sections were obtained using a Leica cutting and grinding system (Leica Microtome, Wetzlar, Germany). The sections were observed under a fluorescence microscope (Penguin 600CL, Pixera) before staining. Then, the sections were stained with 1.2% trinitrophenol and 1% acid fuchsin (Van Gieson staining) and examined under a standard light microscope (Leica LA Microsystems, Bensheim, Germany) equipped with a digital image capture system (Penguin 600CL, Pixera). Bone and material were measured using a digital image analysis system (Image-Pro Plus software, Silver Spring, USA). Bone and material volumes were calculated based on Van Gieson staining and compared statistically.

#### Statistical analysis

Quantitative data are presented as the mean ± standard deviation. One-way ANOVA and least significant difference (LSD) t test were performed for statistical analysis using PASW Statistics 19.0 software (SPSS Inc., Chicago, USA). Differences were considered significant if the P-values were less than 0.05 (^*^P < 0.05). GraphPad Prism 6.0 software (GraphPad Software Inc., La Jolla, USA) was used to plot graphs.

## Results

### Scanning electron microscope and energy spectrum analysis of β-TCP granules

**Figure 3:**
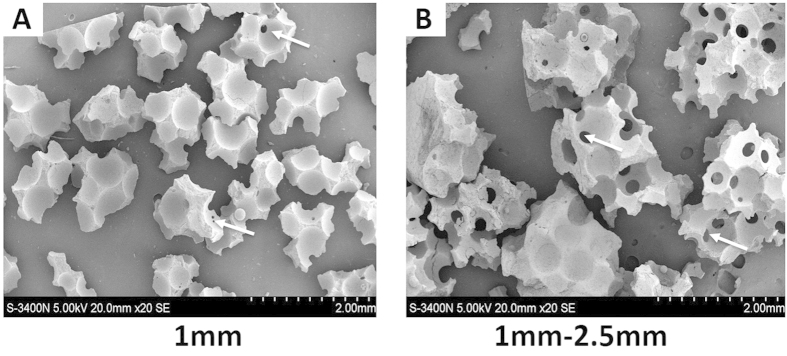
Scanning electron micrograph results for the β-TCP granules. Scale bars: 20×. White arrows indicate interconnections.

**Figure 4:**
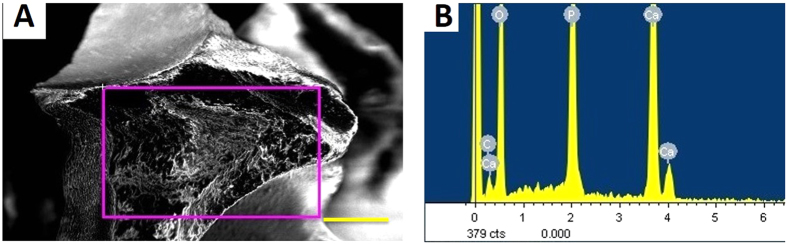
Energy spectrum analysis images. (**A**) Shows the region of interest (ROI) chosen (the purple rectangle). Scale bar: 150 μm.

The porous structure of β-TCP was observed and measured by scanning electron microscopy (SEM) (Fig. 3). The elemental composition and weight percentage of the β-TCP granules were determined by energy spectrum analysis (HORIBA EMAX, EX-450, Japan) (Fig. 4). Table 2 shows the elemental composition and weight percentage of the different elements in the β-TCP granules. The two sizes of β-TCP granules had identical elemental compositions and weight percentages. The resulting Ca/P ratio was 1.68, close to the value of native bone. This ratio ensured that the β-TCP granules provided sufficient and appropriate calcium and phosphorus levels for neo-bone formation.

### Estimation of BMSCs

**Figure 5:**
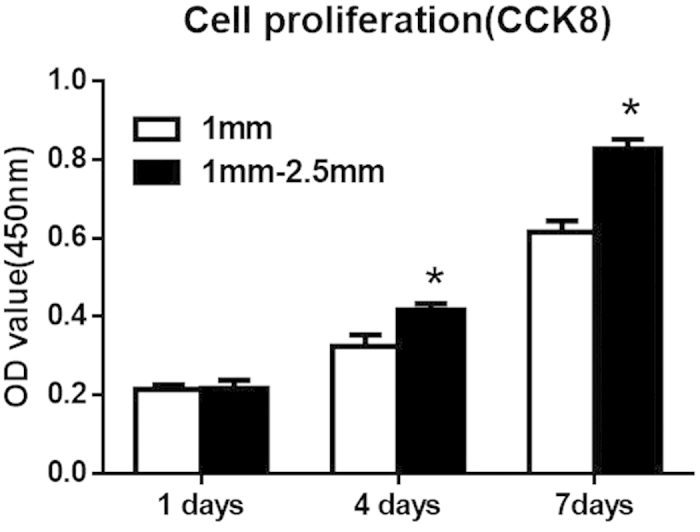
Measurement of BMSC proliferation by the CCK-8 assay after 1, 4, and 7 days of incubation. For each group, n = 3; asterisks (*) indicate statistical significance compared with group (**A**) (1 mm β-TCP granules), P < 0.05.

Cell proliferation was evaluated by the CCK-8 assay. Proliferation increased with incubation time in both group A (1-mm β-TCP granules) and group B (1–2.5 mm β-TCP granules) (Fig. 5). After 1 day of incubation, cell proliferation was nearly identical in groups A and B (P > 0.05). Cell proliferation was significantly greater in group B (1–2.5 mm β-TCP granules) than in group A (1 mm β-TCP granules) at 4 and 7 days (P < 0.05). These results indicate that β-TCP granules can improve BMSC proliferation and that granules with a size of 1–2.5 mm promote greater proliferation of BMSCs.

### Glucose consumption

**Figure 6:**
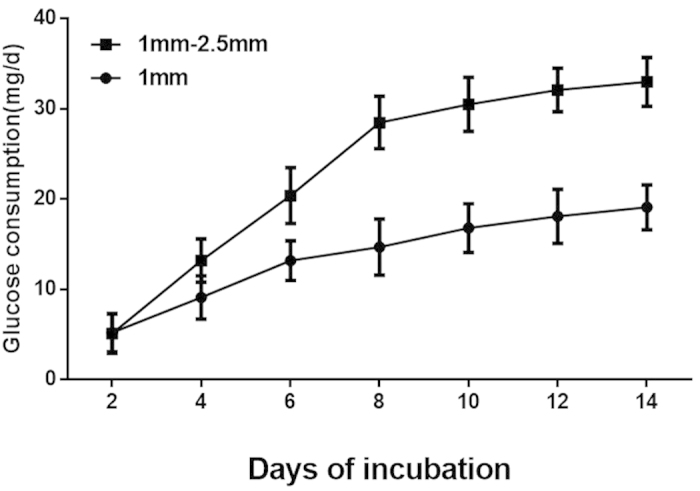
The daily glucose consumption of the cell/scaffold constructs with different granules sizes. Consumption increased gradually in both groups. Group (**B**) (1–2.5 mm β-TCP granules) exhibited higher glucose consumption at all time points than group (**A**) (1 mm β-TCP granules) (P < 0.05) except for day 2.

Glucose consumption was measured as a marker of BMSCs metabolic activity and the quantity of cells on the β-TCP granules. The daily D-glucose consumption rate increased steadily with time in all groups (Fig. 6). Significantly higher glucose consumption was observed in group B (1–2.5 mm β-TCP granules) compared to group A (1 mm β-TCP granules) at all the time points except for day 2 (P < 0.05). In group B, glucose consumption increased rapidly in the first 7 days and then increased slowly during the next 7 days of incubation. In group A, glucose consumption increased relatively gradually during the entire culture period.

### ALP activity

**Figure 7:**
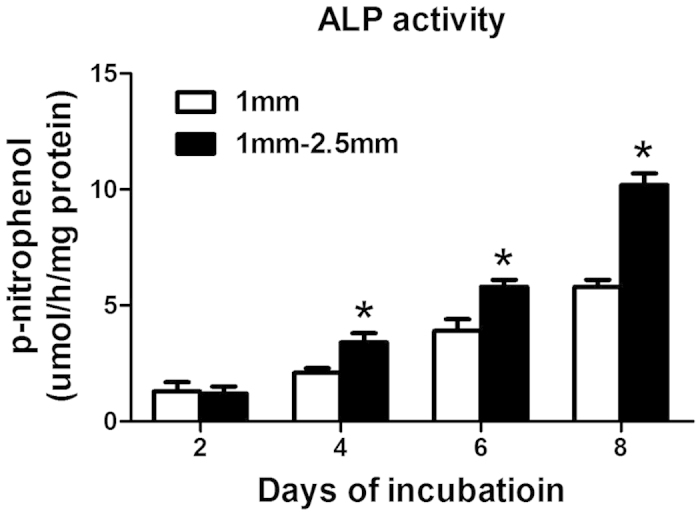
Analysis of ALP activity by a colorimetric assay at different time points. n = 3 for each group, asterisks (*) indicate statistical significance compared with group (**A**) (1 mm β-TCP granules) (P < 0.05).

Intracellular ALP activity was measured at 2, 4, 6 and 8 days of incubation (Fig. 7). The ALP activity increased from day 2 to day 8 in all groups. The ALP activity was higher in group B (1–2.5 mm β-TCP granules) than in group A (1 mm β-TCP granules), but the difference was not significant (P > 0.05) at 2 days of incubation.

### Quantitative real-time PCR

**Figure 8:**
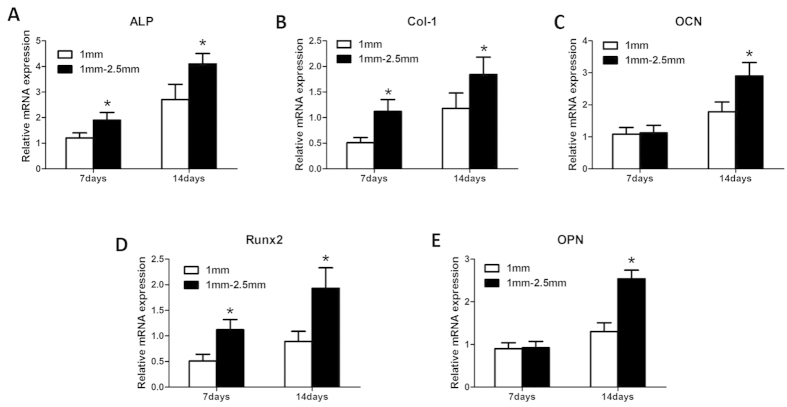
Relative mRNA expression of ALP (**A**), Col-1 (**B**), OCN (**C**), Runx2 (**D**) and OPN (**E**). n = 3 for each group, asterisks (*) indicate statistical significance compared with group (**A**) (1 mm β-TCP granules) (P < 0.05).

The mRNA levels of osteogenic differentiation markers (ALP, COL-1, OCN, Runx2, and OPN) were quantified using RT-PCR to determine which β-TCP granules resulted in the greatest improvement in osteogenic gene differentiation. The expression levels of genes encoding the bone-related proteins ALP, COL-1, and Runx2 were much higher in group B (1–2.5 mm β-TCP granules) than in group A (1 mm β-TCP granules) at 7 and 14 days (P < 0.05) (Fig. 8A,B,D). The gene expression levels of OCN and OPN were significantly higher in group B (1–2.5 mm β-TCP granules) than in group A (1 mm β-TCP granules) at 14 days (P < 0.05), but there was no significant difference at 7 days (Fig. 8C,E) (P > 0.05). These results demonstrate that β-TCP granule scaffolds promote the osteogenic differentiation of BMSCs and that group B (1–2.5 mm β-TCP granules) exhibited greater osteogenic gene differentiation.

### Western blot analysis

**Figure 9:**
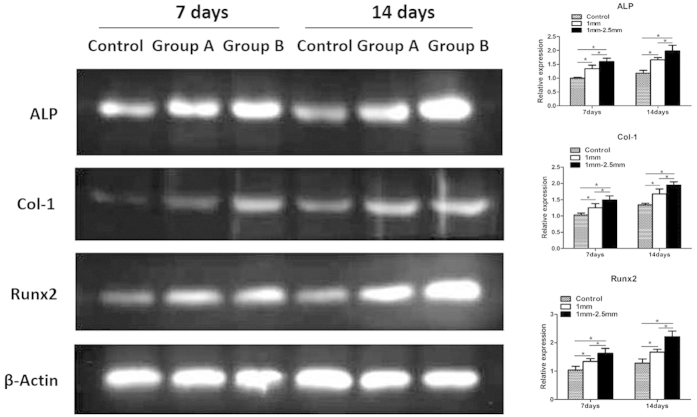
Western blot and semiquantitative analysis of ALP, Col-1, and Runx2 expression in BMSCs cultured with or without β-TCP granules for 7 and 14 days. n = 3 for each group, asterisks (*) indicate statistical significance (P < 0.05).

The expression of osteogenesis-related proteins in BMSCs cultured with or without β-TCP granules is shown in Fig. 9. At 7 and 14 days of incubation, the expression levels of ALP, Col-1 and Runx2 were much higher in the experimental groups (group A and group B) than in the control group (both P < 0.05). However, Col-1 expression was slightly higher in the experimental group than in the control group at 7 days (P < 0.05) and was much higher at 14 days. The expression levels of ALP, Col-1 and Runx2 were significantly higher in group B (1–2.5 mm β-TCP granules) than in group A (1 mm β-TCP granules) (P < 0.05). These results indicated that the β-TCP granule material promotes BMSC osteogenesis and that 1–2.5 mm β-TCP granules have a greater promoting effect on osteogenesis than 1 mm β-TCP granules.

### Fluorochrome labelling

**Figure 10:**
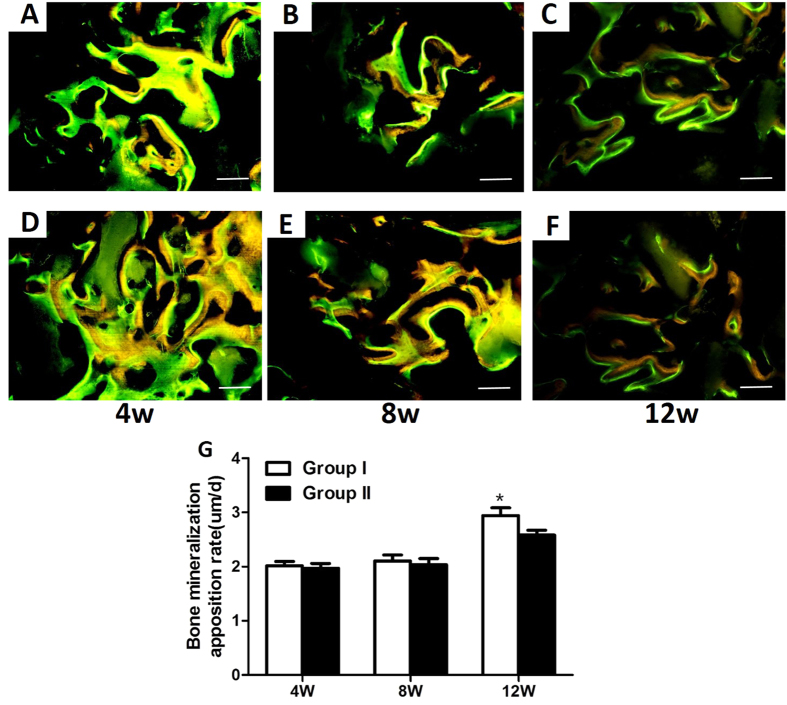
Fluorochrome labelling of regenerated bone in osteo-regenerators at 4 weeks (**A,D**), 8 weeks (**B,E**) and 12 weeks (**C,F**) post-operation in group I (**A–C**) and group II (**D–F**). (**G**) Quantitative analysis results at different time points post-operation. Scale bar: 50 μm (white) (asterisks (*) indicate statistical significance; P < 0.05).

Fluorescent labelling was evaluated in group I and group II (Fig. 10). Green lines indicate the deposition of new calcification with tetracycline, whereas the yellow lines indicate the newly deposited calcification with calcein. The interval between the two lines represents the speed of new bone formation. Quantitative analysis of the fluorochrome marker intervals revealed that calcification deposition was significantly higher in group I than in group II at 12 weeks post-operation. At 4 and 8 weeks, there was no significant difference between group I and group II (P > 0.05) (Fig. 10). Furthermore, both groups I and II exhibited increased calcification deposition throughout the study.

### Micro-CT evaluation

**Figure 11:**
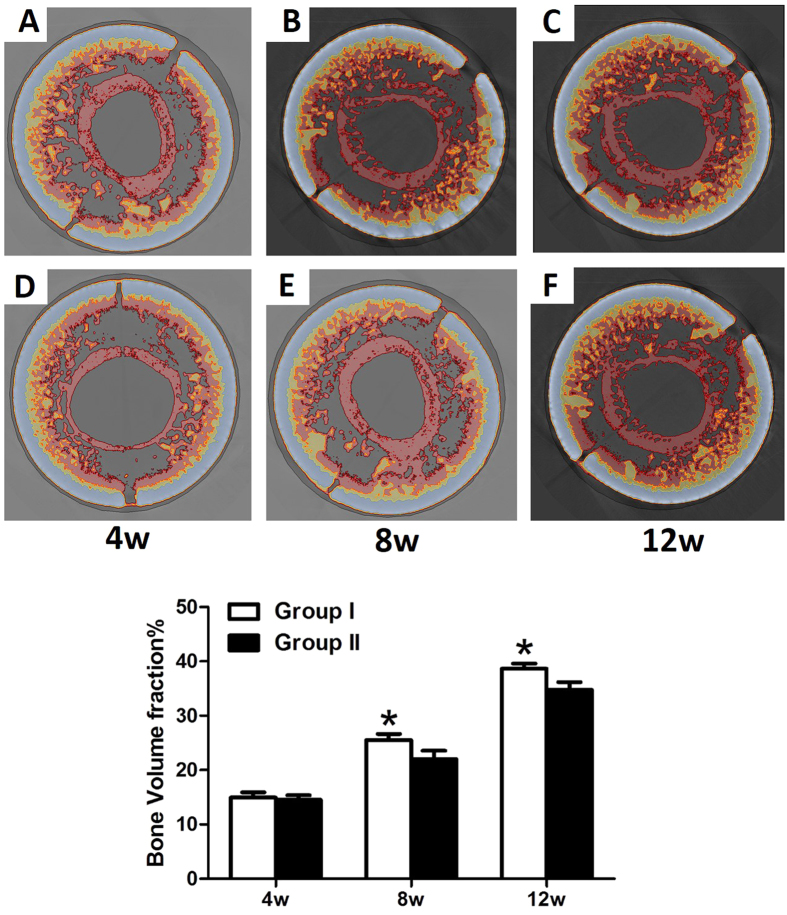
Cross section of the micro-CT images of the osteo-regenerators at 4 weeks (**A,D**), 8 weeks (**B,E**) and 12 weeks (**C,F**) post-operation in group I (**A–C**) and group II (**D–F**). (**G**) Results of the quantitative analyses at different time points post-operation. (asterisks (*) indicate statistical significance. P < 0.05).

Micro-CT was performed to analyse the 3D structure of the osteo-regenerators. Additionally, reconstructed 3D stereoscopic pictures of osteo-regenerators were obtained and analysed at 4, 8 and 12 weeks after implantation (Fig. 11). The grey components are the titanium alloy shields of the osteo-regenerators. The red circle in the middle of the osteo-regenerator is the femur of rabbit, and the other red components are newly formed bone. The yellow parts in the osteo-regenerator are the remaining β-TCP granule materials. Quantitative measurements obtained from the micro-CT data were utilized to analyse the new bone formation in the osteo-regenerator, as indicated by the bone volume fraction (bone volume/total volume of the total osteo-regenerator, BV/TV). Quantitative volumetric analysis revealed that the bone volume fraction in the osteo-regenerators increased significantly in both group I and group II during the study. At 4 weeks after the operation, the bone volume fraction was higher in group I (14.955 ± 0.953%) than in group II (14.534 ± 0.832%), but the difference between the two groups was not significant (P > 0.05). Bone formation was significantly higher in group I than in group II at 8 and 12 weeks (P < 0.05).

### Histological examination of new bone formation and material degradation

**Figure 12:**
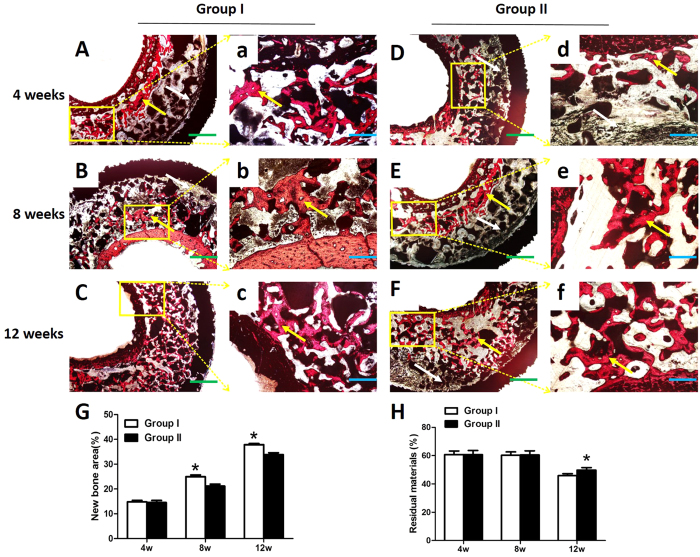
(**A–F,**a-f) Van Gieson staining of histological sections and (**G,E**) quantitative analysis of the new bone and residual material in the osteo-regenerators at 4, 8 and 12 weeks post-operation. The yellow arrow indicates newly formed bone. The connective tissue is marked by a white arrow. Asterisks (*) indicate statistical significance, P < 0.05. Scale bar: 50 μm (blue), 20 μm (green).

Histological analysis was performed by Van Gieson staining to assess osteogenesis in the osteo-regenerators at 4, 8 and 12 weeks after implantation (Fig. 12). Image-Pro Plus 6.0 software (Media Cybernetics, US) was used to calculate the area of new bone. No inflammatory reaction was observed at either time point in the two groups. Four weeks after implantation, moderate regenerated bone tissue (yellow arrow) was observed around the β-TCP granules near the shaft of the femur. The new bone volume did not differ significantly in group I (14.755 ± 0.653%) compared to group II (14.534 ± 0.832) (P > 0.05). Abundant connective tissue (white arrow) growing across the holes in the titanium shields into the osteo-regenerator was observed. At 8 weeks, more newly formed bone was observed in the two groups. The percentage of new bone formation (bone area/total area) in group I was 24.929 ± 0.724%, much higher than that in group II, in which 21.130 ± 0.821% of the porous regions was filled with bone tissue. Twelve weeks after implantation, the osteo-regenerators in group I and group II both had an abundance of newly formed bone tissue. Histomorphometric analysis revealed that the amount of new bone regeneration in group I (37.846 ± 0.457%) was markedly higher than that in group II (33.852 ± 0.852). Both groups exhibited a rapid increase in new bone formation from 4 weeks to 12 weeks.

Material degradation was also observed in this work. The β-TCP granules degraded slowly in both groups until 8 weeks post-operation. At 4 weeks post-operation, the percentage of residual material was 60.686 ± 2.636% in group I and 60.701 ± 2.942% in group II (P > 0.05). In both groups, the percentage of residual material at 8 weeks was nearly identical to that at 4 weeks. However, the β-TCP granules degraded rapidly 8 weeks after implantation. The percentage of residual material in group I was 45.849 ± 1.414%, much lower than in group II (49.753 ± 1.824%) (P < 0.05).

## Discussion

Due to limitations in reconstructing critical bone defects^37^,^38^,^39^, bioreactors for bone tissue engineering have become an alternative strategy for skeletal reconstruction^40^,^41^,^42^,^43^. None of the *ex vivo* tissue engineering procedures developed over more than a decade have reach the clinical routine^44^,^45^,^46^,^47^. Consequently, *in vivo* bioreactors have been developed that use the body itself as reactor to form the required bone substitute with the help of bioactive scaffolds^48^. Scaffolds with good osteoconductivity, biocompatibility, and biodegradability are also indispensable for bioreactor construction^49^. Thus, identifying an ideal site and an optimal scaffold to construct an *in vivo* bioreactor is of great importance^50^.

The porous architectural characteristics of the β-TCP scaffold material profoundly affected post-implantation osteogenesis, enabling cell survival and tissue growth in porous biomaterials^30^,^50^. In this *in vitro* study, the osteogenesis promoted by 1 mm and 1–2.5 mm β-TCP granules with pores and interconnections was evaluated. CCK-8 assay results revealed that the β-TCP granules with a diameter of 1–2.5 mm had a greater enhancing effect on BMSC proliferation than those with a diameter of 1 mm. Glucose consumption assays indicated that 1–2.5 mm β-TCP granules resulted in increased metabolic activity, indirectly indicating the quantity of cells. Additionally, 1–2.5 mm β-TCP granules also resulted in increased ALP activity.

In addition to cell proliferation, cell differentiation is critical for bone regeneration. Real-time PCR analysis revealed that the expression levels of the osteogenic genes ALP, Col-1 and Runx2 were higher in group B than in group A at 7 and 14 days (Fig. 8A,B,D, respectively). The gene expression levels of OCN and OPN were significantly higher in group B (1–2.5 mm β-TCP granules) than in group A (1 mm β-TCP granules) at 14 days (P < 0.05) but did not differ significantly between the two groups at 7 days (Fig. 8C,E) (P > 0.05).

The expression of osteogenesis-related proteins in BMSCs cultured with or without β-TCP granules was assessed at each time point. The expression of ALP and Runx2 was significantly higher in groups A and B than in the control group. Col-1 expression was slightly higher in the experimental groups than in the control group at 7 days (P < 0.05) but was much higher at 14 days. The expression levels of ALP, Col-1 and Runx2 were significantly higher in group B (1–2.5 mm β-TCP granules) than in group A (1 mm β-TCP granules) (P < 0.05). The 1–2.5 mm β-TCP granules may have had a greater effect on osteogenesis due to their greater size variation and greater contact area for osteogenesis.

These results indicated that β-TCP granules with sizes of 1 mm and 1–2.5 mm with a porous structure and interconnections improved BMSC proliferation and osteogenesis *in vitro*. Furthermore, the β-TCP granules with diameters of 1–2.5 mm resulted in greater osteogenesis compared with the 1 mm granules.

In the *in vivo* experiment, two different methods were used to establish the osteo-regenerators. In group I, we drilled two holes that perforated the middle of the femoral shaft. By contrast, in group II, the periosteum of the femur was incised. During the early stage after implantation, connective tissue grew into and attached to the osteo-regenerator, whereas newly formed bone grew near the femur, which was positioned in the centre of the osteo-regenerators. During the late stage, the new bone became abundant, and the connective tissue was replaced by newly formed bone. Fluorescent labelling indicated that the calcification deposition was significantly faster in group I than in group II at 12 weeks after implantation. However, there was no marked difference among group I and group II at 4 weeks and 8 weeks. Micro-CT was performed to evaluate the newly formed bone volume. Quantitative analysis demonstrated that the new bone formation was notably higher in group I than in group II at 8 weeks and 12 weeks post-operation. By contrast, at 4 weeks, there was no significant difference in new bone formation between the two groups. Histological examination and quantitative histological analysis were performed to further assess the osteo-regenerator. Quantitative measurements indicated that both groups had favourable bone generation, but the percentage of new bone was much higher in group I than in group II at 8 weeks and 12 weeks after implantation. No obvious difference was observed between the two groups at 4 weeks, consistent with the results of the fluorescent label and micro-CT analyses. The material degradation analysis demonstrated that degradation occurred slowly in both groups until 8 weeks post-operation. At 12 weeks, the degradation rates were higher in both groups, with faster degradation in group I than in group II (P < 0.05).

Several elements are required for an *in vivo* bioreactor: (1) osteoinductive proteins that accelerate the differentiation of stem cells into osteogenic cells; (2) blood vessels for the delivery of stem cell progenitors; (3) an osteoconductive scaffold; and (4) a complete microcosm for osteogenesis that allows cell populations to grow into bone^51^,^52^. The distinguishing features of the β-TCP granule scaffolds are its interconnecting open multi-porosity holes, which facilitate the in-growth of blood vessels for supplying nutrients to the ingrown cells and newly formed bone^30^,^53^. The microporosity of the scaffold granules greatly expanded the surface area of the materials, enabling the attachment of more cells and growth signals and wetting by plasma and tissue fluids to enhance new bone formation. The multi-pore system of the osteo-regenerators allowed the entry of growth factors and the in-growth of bone-forming cells and connective tissue. The large holes in the titanium shields and the New Zealand rabbit femur, as well as the small holes in the β-TCP granules, permitted communication and exchange between the bone and soft tissue microenvironments of the osteo-regenerators. The combination of these factors promoted the multicentred sources of osteogenesis in the osteo-regenerator.

The *in vivo* experiment demonstrated that both methods of constructing the osteo-regenerators enhanced bone formation. Compared with group II, group I possessed exhibited bone generation facilitated by the entry of intrinsic cellular and signalling elements and bone growth factors through the holes in the femoral shaft of the New Zealand rabbits. In group I, the marrow cavity of the femur provided the ingrown cells, signalling elements and bone-growth factors needed for the entire osteo-regenerator. By contrast, in group II, the necessary substances could only be supplied by the periosteum, which was limited compared to the marrow cavity. Key benefits of the *in vivo* approach are the reduction of the manipulation of cells *in vitro* and exploitation of the body’s own regenerative capacity for the regrowth and development of bone tissues, thus creating a more physiological niche for new bone formation.

This study demonstrated that the β-TCP granules promoted osteogenesis *in vitro*. The new osteo-regenerators, i.e., *in vivo* bioreactors, constructed using the two methods described above facilitated new bone formation. However, our work has limitations. The precise reason why the β-TCP granules with sizes of 1–2.5 mm were superior to 1 mm granules remains unknown, and further study is needed. The relationship between bone generation ability and the large holes in the titanium shields and femur remains to be elucidated. Moreover, the defect reconstruction ability of this new bone harvested from the osteo-regenerator has not yet been studied. These questions will be addressed in future research.

## Conclusion

The *in vitro* study demonstrated that β-TCP granules scaffolds with sizes of 1 mm and 1–2.5 mm can improve the proliferation of BMSCs and promote the expression of osteogenic genes and osteogenesis-related proteins. Additionally, the 1–2.5 mm granules scaffolds exhibited superior function compared to the 1 mm β-TCP granules. Furthermore, the *in vivo* study demonstrated that the osteo-regenerators with 1–2.5 mm β-TCP granules facilitated new bone formation without the application of exogenous cells and growth factors and that the osteo-regenerator with two holes that perforated the femur resulted in greater bone formation compared with the osteo-regenerator using a periosteal incision. This new technology may provide a treatment for bone defects.

## Additional Information

**How to cite this article**: Gao, P. *et al.* Beta-tricalcium phosphate granules improve osteogenesis *in vitro* and establish innovative osteo-regenerators for bone tissue engineering *in vivo. Sci. Rep.* **6**, 23367; doi: 10.1038/srep23367 (2016).

## Figures and Tables

**Table 1:** Primers used in real-time PCR.

Target gene	Forward primer (5′-3′)	Reverse primer (5′-3′)
ALP	TTGGGCAGGCAAGACACA	GAAGGGAAGGGATGGAGGAG
Col-1	GACATGTTCAGCTTTGTGGACCTC	GGGACCCTTAGGCCATTGTGTA
OCN	ACCATCTTTCTGCTCACTCTGCT	CCTTATTGCCCTCCTGCTTG
Runx2	GAACCAAGAAGGCACAGACAGA	GGCGGGACACCTACTCTCATAC
OPN	TACGACCATGAGATTGGCAGTGA	TATAGGATCTGGGTGCAGGCTGTAA
GAPDH	TGCTGGTGCTGAGTATGTGGT	AGTCTTCTGGGTGGCAGTGAT

**Table 2:** The composition and weight percentage in β-TCP granules.

Element	Weight percentage	Atom percentage
C	5.64	8.89
O	50.50	65.78
P	16.37	11.09
Ca	27.49	14.24
Gross	100	100
